# WATER TREATMENT: Sweeteners Persist in Waterways

**Published:** 2009-10

**Authors:** Rebecca Kessler

**Affiliations:** **Rebecca Kessler**, based in Providence, Rhode Island, is a senior editor at *Natural History* and writes about science and the environment for various publications. She is a member of the National Association of Science Writers and the Society of Environmental Journalists

Artificial sweeteners are widespread in European sewage treatment plant effluent, waterways, groundwater, and even drinking water, a growing body of research demonstrates. One of the latest studies, published in the July 2009 issue of *Analytical and Bioanalytical Chemistry*, presents data on four common sweeteners found in German water and demonstrates the persistence of these additives. Two, acesulfame and sucralose, were remarkably resistant to treatment by conventional sewage treatment plants as well as by a more advanced soil aquifer treatment plant, report environmental engineer Marco Scheurer and colleagues from the Water Technology Center in Karlsruhe.

In samples taken from four German rivers, concentrations of ace‐sulfame exceeded 2 μg/L, whereas concentrations of sucralose, cyclamate, and saccharin were an order of magnitude lower. Coauthor Frank T. Lange, an analytical chemist, notes that a person would have to drink 2–3 L of water with sweetener concentrations similar to those of the German rivers every day for years before they would consume the amount contained in a single sweetener tablet (the concentrations detected in water were well below human taste thresholds). Three other sweeteners—aspartame, neotame, and neohesperidin dihydrochalcone—were not detected in any of the samples.

The findings echo those of four recent studies documenting artificial sweeteners in sewage treatment plant effluent and waterways throughout Europe. Preliminary results from two separate studies—one unpublished and one accepted 11 September 2009 for publication in *Marine Chemistry*—also show sucralose in Arizona wastewater treatment plant effluent and several downstream rivers, as well as in coastal and Gulf Stream waters off the southeastern United States.

Acesulfame and sucralose, which is sold in the United States as Splenda®, have proven to be the most commonly found and resilient sugar substitutes. They are added to a wide variety of foods, beverages, pharmaceuticals, and toiletries, and they pass through the human body virtually unchanged. Cyclamate and saccharin are much less persistent. Cyclamate has been banned in the United States since 1970 as a possible human carcinogen, but the Food and Drug Administration is considering reapproval. And although saccharin has been found to cause cancer in rats, it is considered safe for human consumption.

All the sources interviewed for this article agree there’s little risk that acesulfame and sucralose in drinking water will cause human health problems. The implications for the aquatic environment are less clear, however. Because these sweeteners have been classified as safe for human consumption, they have undergone virtually no environmental testing. Yet the remarkable persistence of acesulfame and sucralose gives some experts pause, given that environmental concentrations will likely rise over time with continued consumption.

Henrik Kylin, an environmental chemist at the Norwegian Institute for Air Research and a coauthor of a study on sucralose presented at the Society of Environmental Toxicology and Chemistry Europe 17th Annual Meeting in 2007, points out that sucralose mimics sucrose, a structurally similar molecule involved in biological functions from the regulation of genes related to photosynthesis to feeding cues in zooplankton. “If those [functions] are affected, you may end up with serious ecosystem effects,” he says.

Kylin is also concerned by the finding, reported in the October 2006 issue of *Plant, Cell & Environment*, that sucralose at least partially inhibited sucrose transport in sugarcane. “There are very many vascular plants in the aquatic ecosystem,” he says, “and if they are [similarly] affected, it would affect very many other organisms.”

Rosa Krajmalnik‐Brown, an assistant professor of environmental biotechnology at Arizona State University, has been working for more than two years to identify a microorganism that can degrade sucralose and hasn’t found one yet—although she says she’s not giving up. Still, there may be a bright side to sweeteners’ persistence: Scheurer and other researchers have proposed using them as markers for detecting wastewater spills—a welcome finding for scientists who have long sought a failsafe marker.

